# Wearable Technologies for Health Promotion and Disease Prevention in Older Adults: Systematic Scoping Review and Evidence Map

**DOI:** 10.2196/69077

**Published:** 2025-06-24

**Authors:** Yue Sun, Ji Chen, Mengmeng Ji, Xiaomei Li

**Affiliations:** 1 School of Nursing Health Science Center Xi'an Jiaotong University Xi'an China; 2 Mianyang Research Institute of Traditional Chinese Medicine Mianyang Hospital of Traditional Chinese Medicine Mianyang China; 3 Department of Endocrinology Peking University First Hospital Peking University Beijing China

**Keywords:** wearable technology, older adults, scoping review, evidence map, systematic review

## Abstract

**Background:**

The demand for wearable technologies has surged in recent years, demonstrating remarkable potential, especially in health promotion. However, there is currently a lack of clarity about the types and roles of wearable devices in health care of older adults.

**Objective:**

This review aims to provide a comprehensive overview and categorize the current research conducted with wearable devices for health promotion and disease prevention in older adults.

**Methods:**

We conducted a systematic literature review using the PRISMA-ScR (Preferred Reporting Items for Systematic Reviews and Meta-Analyses extension for Scoping Reviews) framework and synthesized the results. A total of 6 databases were searched to identify wearable devices reported in studies from inception to July 28, 2024. Titles, abstracts, and full texts were independently screened by 2 reviewers. Any discrepancies were resolved by a third reviewer when necessary. The types of results from relevant studies were systematically mapped into predefined categories.

**Results:**

Based on the inclusion criteria, 109 studies were included. The most commonly reported health targets of wearable devices were mobility, mental health, fall-related, arrhythmia detection, activity recognition, disease diagnosis, and sleep monitoring. Most studies were application design and observational study, and in European countries and the United States, 51 studies of the participants were healthy. The most popular anatomical landmarks for wearable placement were the wrist, waist, and chest. Two evaluation approaches for wearable devices were used: performance metrics in controlled settings and real-world assessments with end users. The opportunities presented by wearable devices are countered by multiple challenges, including data availability and reliability, technical limitations, utility and user acceptance, cost, security and privacy, performance gaps, and challenges.

**Conclusions:**

Wearable devices hold great promise for promoting health in older adults, but several hurdles remain for full adoption. A broader and more diverse group of older adults is needed to identify the most beneficial wearables and to optimize the technology. Further studies are required to statistically synthesize real-world performance and evaluation results. We hope that this review will serve as a valuable reference for the development of wearable devices in older adults.

## Introduction

Wearable health technology refers to electronic devices that are close to or worn on a body part or embedded in clothing or accessories, which detect and collect data through retrieval methods [1]. It uses the miniaturization of sensors and the integration of network connectivity and predictive analytics to automatically capture, transmit, and analyze biometric information [2]. These intelligent devices not only play a role in disease diagnosis [3,4] and treatment but also promote health improvement [5]. They actively record physiological parameters and monitor metabolic status, providing valuable information for personal health management and clinical care [6]. Wearable devices play a crucial role in tracking health conditions, remote health monitoring, and ongoing treatments [7]. By providing real-time data, these devices enable early detection of health issues, promote independent living, and encourage healthy behaviors [8].

With global aging, the share of the global population aged 65 years or older is projected to rise from 10% in 2022 to 16% by 2050 [9], older adults are more susceptible to chronic diseases and functional decline and face critical health challenges that necessitate a significant shift in lifestyle and health care needs [10]. These individuals are at high risk for many symptoms that studies with wearable devices attempt to monitor. On one hand, wearable technologies will play a significant role in advancing precision by enabling the precise measurement of clinically relevant parameters [11]. For instance, continuous health tracking through wearable devices, such as heart rate, blood pressure, and sleep monitoring, provides the detailed, real-time information needed to manage chronic conditions proactively and enable timely medical interventions [12-14]. On the other hand, wearable technologies integrate advanced algorithms and machine learning to precisely address users’ diverse needs, offering more personalized and effective solutions [15]. Applied research to explore applying machine learning techniques using the body signals for elderly care has been growing over the last decade. Among the areas that have garnered researchers’ attention are fall detection [16,17], vital sign monitoring and prediction [18], and activity recognition [19,20]. In addition, these devices promote independence by assisting with daily activities through reminders for medication, appointments, and hydration [21-23]. However, the current applications of wearable health technology primarily focus on certain members and groups within the general population, and there are numerous issues for special populations [24], especially older adults, such as the lack of physical presence and face-to-face contact, as well as concerns about the ethics and acceptability of new technologies [25]. This review found that several key factors influence the acceptance and use of wearable devices by older adults, including intrinsic and extrinsic motivation for device use, ease of use, device purpose, and perceived added value to the user’s life [26]. Addressing the unmet care and technical support needs of an aging population and designing services and solutions around the needs or desires of older adults, is becoming an urgent public health priority [25,27]. To effectively meet these needs, it is crucial to identify and understand existing technologies. To date, there is limited recent evidence synthesis regarding wearable devices for older adults.

The research on the use of wearable devices is increasing, with studies expanding in areas such as palliative care [28], cardiovascular medicine [29], mental health [30] and chronic disease self-management [31]. However, evidence is still lacking regarding the wider use of wearable devices among older adults. A scoping review, as a preliminary assessment of the potential size and scope of available research on a topic, aims to identify the nature and extent of research evidence [32]. Evidence mapping is a useful methodology to provide an overview of available research about broad knowledge areas. We used evidence mapping to represent the volume of work in different content areas, and the maps can provide an organized and understandable presentation of a large body of research [33]. We conducted a systematic scoping review of existing research and created an evidence map. This systematic review responds to the following research questions:

- What are the basic characteristics of the published wearable devices?
- What are the health targets of wearable devices used by older adults?
- Which technologies are being used in wearable devices for older adults?
- How is current research evaluating wearable devices for older adults?
- What are the challenges and opportunities associated with wearable devices for older adults?

## Methods

### Overview

This scoping review was guided by the framework and principles reported by Arksey and O’Malley [34] and in accordance with the PRISMA-ScR (Preferred Reporting Items for Systematic Reviews and Meta-Analyses extension for Scoping Reviews) guideline [35]. A scoping review provides a literature overview by mapping key concepts in the evidence base of the research field, which can be used to inform needs and identify knowledge gaps [36]. PRISMA-ScR checklist for this review is presented in Multimedia Appendix 1.

### Information Sources

A comprehensive search strategy was developed by information and health specialists using a combination of MeSH (Medical Subject Headings) terms and free-text terms. The databases searched were PubMed, Web of Science, CINAHL, Association for Computing Machinery (ACM), IEEE Xplore, and ScienceDirect from inception to July 28, 2024. In addition, relevant studies were identified through a manual search of the reference lists from all available records and conference proceedings obtained in the initial search. The actual strategies listing all search terms used and how they are combined are available in the Multimedia Appendix 2.

### Inclusion Criteria

Eligibility for our scoping review was determined using the PCC (Population, Concept, and Context) framework, which is listed in Textbox 1.

**Inclusion criteria for this scoping review.**

**Population**

• Older people (all participants aged 50 y or older)

• Individuals of any health status (healthy, at risk of disease, or with existing conditions)

**Concept**

• Wearable technologies

**Context**

• Health Promotion and Disease Prevention: described or implemented wearable technologies whose primary focus was to improve health

• Any health target in the context of healthy aging (eg, mobility, mental health, cognition, or disease diagnosis)

• Any nonclinical or community-based settings

**Study type**

• Primary studies with any designs

• Reviews with systematic methodology (eg, systematic reviews)

### Study Selection

We imported the retrieved records into EndNote X9 (Thomson Reuters Scientific LLC) for management. The full texts of the included articles were independently evaluated by 2 reviewers. Disagreements regarding selection and inclusion were resolved through consensus-based decision-making.

### Data Extraction

The data extraction form was based on a previous publication and adapted to the needs of this review on the same topic, and we randomly selected 10 studies to test and refine it. The included studies obtained information on target population, sample size, age, wearable strategies, measurements, main results, and conclusions. The data extraction process was performed by two authors, and inconsistent data were resolved by the third author.

### Data Analysis (Mapping the Evidence)

Data extracted from primary studies were mapped to visually summarize outcome measures identified and coded. We summarized the results using a narrative descriptive synthesizing approach and presented them in tables and figures. The evidence map presents outcome measures by tabulating the type of wearable devices against the domain of the outcome measure. The data extraction form used in this review was piloted and is shown in Multimedia Appendix 3.

## Results

### Included Studies

**Figure 1:**
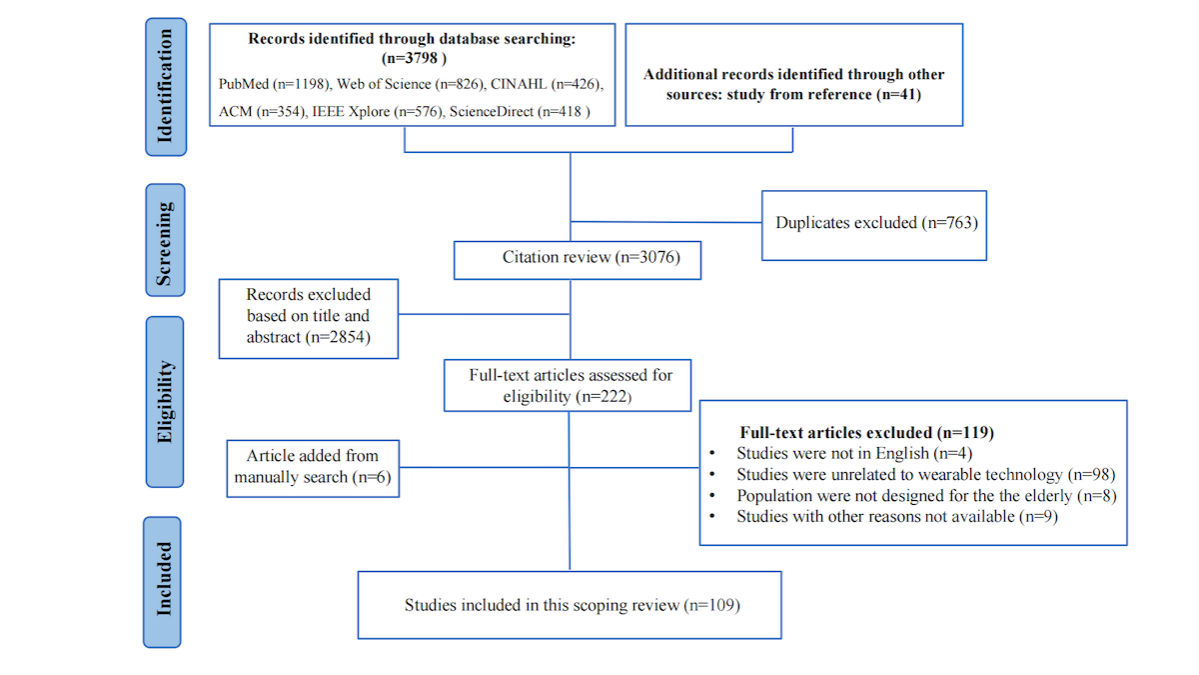
The schematic flow for the selection of the included studies. ACM: Association for Computing Machinery.

The schematic flow for the selection of the studies included is shown in Figure 1. In total, 3798 studies were identified. After removing duplicate citations, 3076 studies remained for the title and abstract screening, of which 222 were considered potentially eligible studies for full-text review. Among them, 109 papers met the inclusion criteria after full-text review [37-125].

### Bibliographic Characteristics of Included Studies

**Figure 2:**
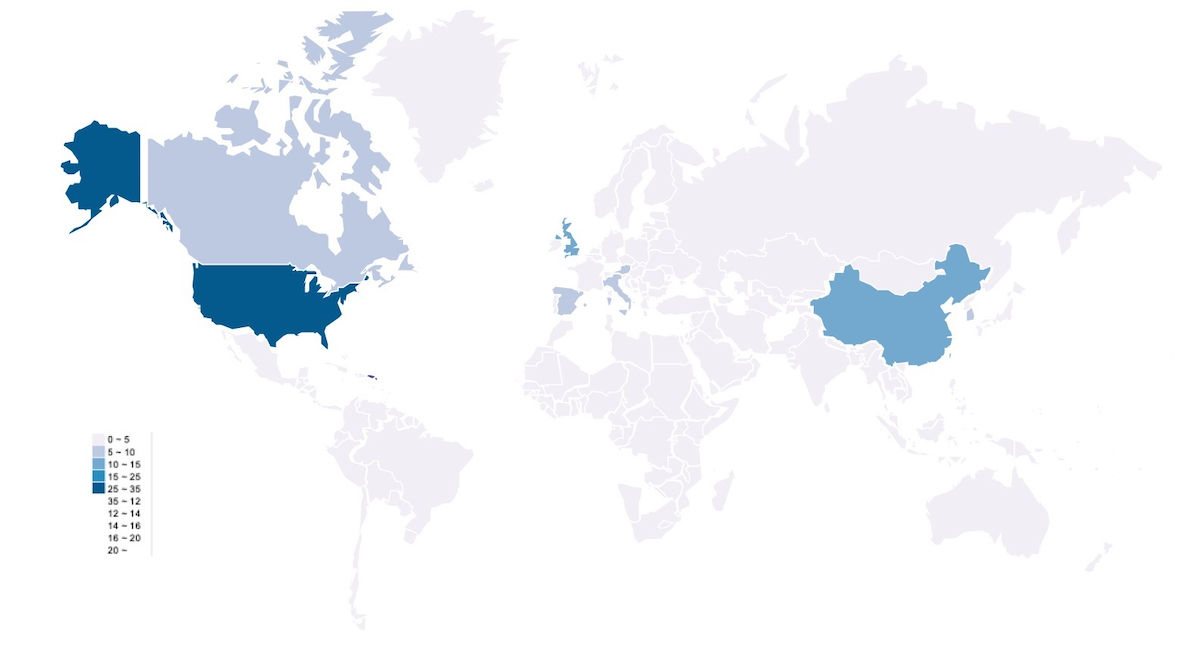
Distribution of the included articles in geographical map.

The number of studies generally increased over time, except for a decline in 2018 (Table 1). They were conducted across 26 countries, with more than half of the studies originating from European countries and the United States (Figure 2 and Multimedia Appendix 4). Most of the included participants mostly did not report any health conditions (51/109, 46.79%), The remaining studies primarily focused on populations with frailty, Parkinson disease, atrial fibrillation, chronic obstructive pulmonary disease (COPD), stroke, and other conditions. The study designs were diverse, the application design studies (38/109, 34.86%) and observational studies (37/109, 33.94%) formed the largest proportion, accounting for nearly two-third of all studies. The characteristics of the included studies are presented in Multimedia Appendix 4.

**Table 1:** Detailed information on all included studies.

Study characteristics		Number of publications, n
**Publication year**		
	2011	2
	2013	3
	2014	6
	2015	6
	2016	7
	2017	9
	2018	14
	2019	12
	2020	10
	2021	11
	2022	11
	2023	13
	2024	5
**Study designs**		
	Application design study	38
	Case-control study	2
	Diagnostic trial	4
	Mixed methods	5
	Observational study	37
	Pilot study	2
	Qualitative study	3
	RCT^a^	11
	Retrospective study	2
	Systematic review	5
**Health status**		
	Health	51
	Frailty	15
	Parkinsons	8
	Atrial fibrillation	6
	Chronic obstructive pulmonary disease	4
	Stroke	4
	Mild cognitive impairment	3
	Obesity	1
	Cancer	2
	Dementia	2
	Depression	2
	Heart disease	2
	Neurological disorders	2
	Cognitively impaired	1
	Diabetes	1
	Diseased gait	1
	Knee osteoarthritis	1
	Other	3

^a^RCT: randomized controlled trial.

### Health Targets of Wearable Devices Used by Older Adults

**Figure 3:**
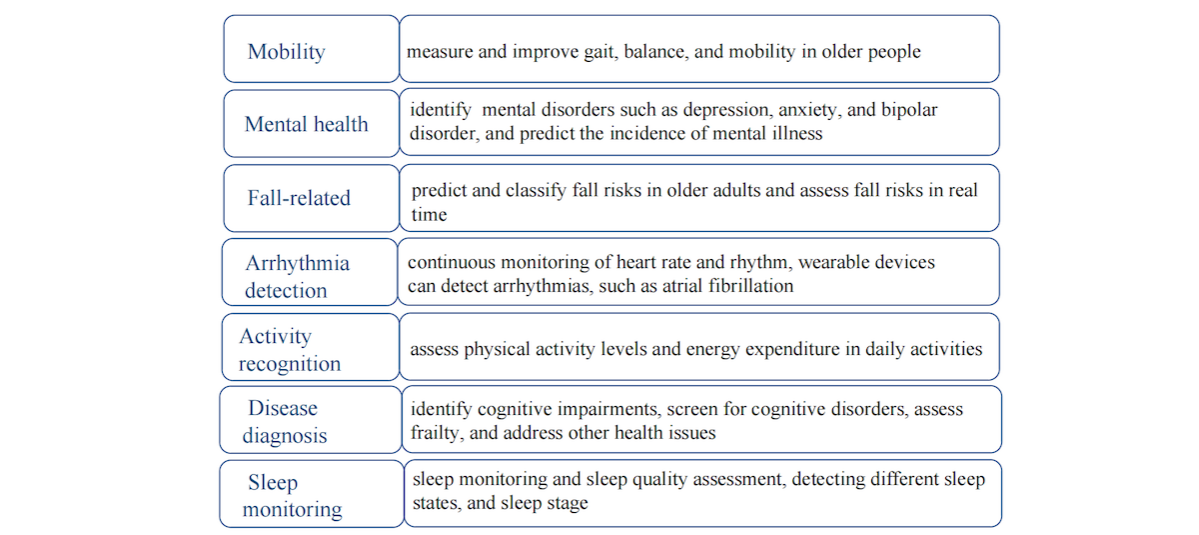
Health targets of wearable devices technologies.

The most commonly reported health targets of wearable devices were (1) mobility (n=23/109), (2) mental health (6/109), (3)fall-related (18/109), (4) arrhythmia detection (10/109), (5)activity recognition (22/109), and (6) disease diagnosis (6/109),and (7) sleep monitoring (8/109) and other conditions. The results are shown in Figure 3.

### Techniques Used in Wearable Devices Used by Older Adults

#### Overview of Wearable Sensor

Wearable techniques comprise devices and sensors that can be attached to the human body for data collection. Wearable sensors consist of accelerometers, photoplethysmography, thermometers, gyroscopes, magnetometers, etc. An overview of wearable sensors is given in Table 2.

**Table 2:** Types of wearable sensors and their applications.

Name of sensor	Purpose	Application
Accelerometer	Measures the acceleration forces acting on it, including gravity. It can detect changes in speed and direction	Used in applications like step counting, motion detection, and orientation sensing
PPG^a^	Detects blood volume changes in the microvascular bed of living tissue	Monitor heart rate, blood oxygen saturation (SpO_2_), and even estimate blood pressure
Thermometer	Measures body temperature	Used in medical settings, homes, and increasingly in wearable devices to monitor body temperature
Gyroscope	Measures the angular velocity or rotation rate of an object	Used in combination with accelerometers in many devices to improve the accuracy of motion tracking
EEG^b^	Measures the electrical activity of the brain	Used in medical diagnostics, neuroscience research, brain-computer interfaces (BCIs), sleep analysis, and mental state monitoring
ECG^c^	Measures the electrical activity of the heart	Used for diagnosing heart conditions, monitoring heart rate, and detecting arrhythmias. Commonly found in clinical settings and wearable health devices
Pressure sensor	Measures the force or pressure applied to it	Used in blood pressure monitoring, industrial processes, and environmental monitoring
Magnetometer	Measures the strength and direction of magnetic fields	Used in navigation systems, compasses, and for detecting magnetic fields in the environment
EMG^d^	Measures the electrical activity produced by skeletal muscles	Used in medical diagnostics, rehabilitation, and in the development of prosthetics
IMU^e^	Combines multiple sensors (typically an accelerometer, gyroscope, and sometimes a magnetometer) to measure and report on a device’s specific force, angular rate, and sometimes the magnetic field surrounding the device	Used in motion tracking, navigation, and stabilization

^a^PPG: photoplethysmography.

^b^EEG: electroencephalography.

^c^ECG: electrocardiography.

^d^EMG: electromyography sensor.

^e^IMU: inertial measurement unit.

#### Placement of Wearable Sensor

**Figure 4:**
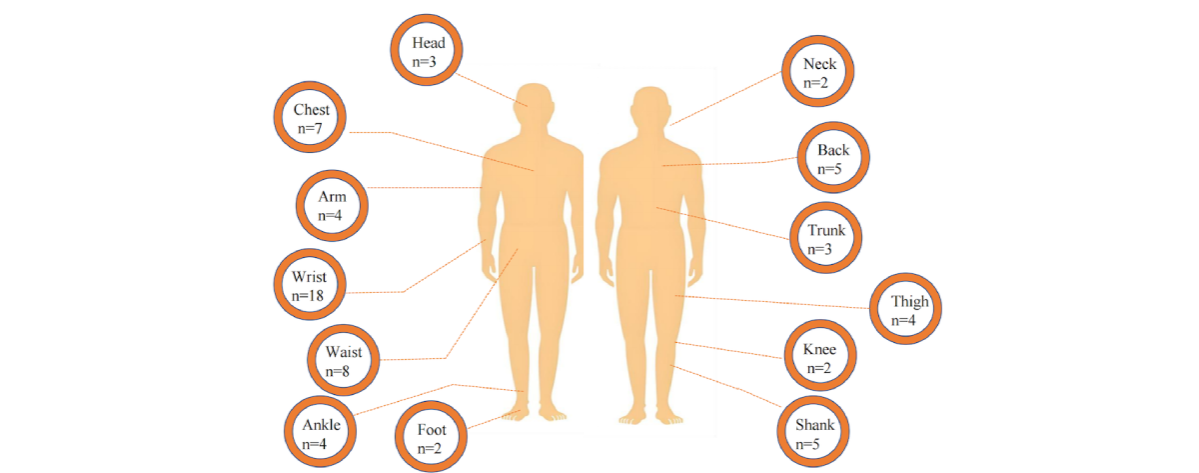
Placement of wearable sensors.

Wearable devices are typically worn on four main areas of the human body: head-mounted, wrist-worn, portable (depending on the specific context), and body-worn. Depending on the specific application requirements, these devices can be positioned at various locations on the body to measure different parameters. Figure 4 illustrated the various positions where sensors are placed for data collection. The analysis showed that the most commonly used placement is the wrist, waist, chest, back, and ankle.

#### Statistical or Algorithms

Table 3 presents the key characteristics of machine learning techniques in research studies, including tree-based models (decision tree, random forest, and gradient boosting), linear models, deep learning neural networks, multilayer perceptrons, long short-term memory networks, and convolutional neural networks). K-nearest neighbors, support vector machines, and others.

**Table 3:** Machine learning techniques research work for wearable sensors.

Category and ML^a^ techniques			Reason for use
**Decision Trees and Ensemble methods**			
	Decision tree	Handles high-dimensional data. Robust to noisy data.	
	Random forest	Robustness through ensemble learning. Handles imbalanced datasets, Suitable for large datasets.	
	Gradient boosting	Improves model performance iteratively. Flexible parameter tuning.	
	XGBoost^b^	Efficient through parallel computation. Optimizes to prevent overfitting.	
**Linear models**			
	Logistic regression	Simple and efficient. Easy to train and optimize.	
	Elastic net regression	Handles multicollinearity. Sparse solution.	
	Ridge regression	Handles overfitting by adding bias.	
	Lasso regression	Performs feature selection by shrinking unimportant features’ coefficients to zero.	
	Linear regression	Basic model for simple linear relationships.	
**Deep learning models**			
	Convolutional neural network	Automatic feature extraction. Suitable for image and signal processing tasks.	
	Multilayer perceptron	Learns nonlinear mappings.	
	Long short-term memory	Handles sequence data effectively.	
	Deep neural network	Handles data from different domains. Customizable for personalized health monitoring.	
**Nearest neighbor algorithms**			
	K-nearest neighbor	Simple and intuitive, suitable for real-time classification tasks.	
**Support vector machines**			
	Support vector machine	Handles high-dimensional data effectively. Robust to nonlinear relationships.	
**Bayesian methods**			
	Naïve Bayesian	Handles uncertainty well.	
**Mixture models**			
	Gaussian mixture models	Captures complex distribution patterns.	

^a^ML: machine learning.

^b^XGBoost: extreme gradient boosting.

### Evaluation Approach of Wearable Devices

In this study, we found two types of wearable devices evaluation approaches: (1) metrics for performance evaluation in a controlled laboratory and clinical setting and (2) evaluations involving end users in real-world settings. Analysis revealed that different studies use a variety of metrics for performance evaluation. Despite variations in machine learning algorithms, the figure showed the evaluation metrics used across 45 studies. In our study, 39.45% (43/109) of included studies used the metrics to assess performance. Sensitivity (29/109, 26.61%), accuracy (23/109, 21.10%), specificity (22/109, 20.18%), precision recall (11/109, 10.09%), and *F*_1_-score (11/109, 10.09%) were the most used metrics among offline evaluation metrics. Other popular offline evaluation metrics are accuracy-related measurements, such as root-mean-square error (3/109, 2.75%), and the mean absolute error (1/109, 0.92%).

In addition to performance testing, there were also 34 studies involving end users that evaluated the effectiveness, safety, user acceptability, usability, adherence, satisfaction, user experience, cost, and satisfaction. Clinical effectiveness (n=22, 20.18%) and acceptability (n=17, 15.60%) were the most commonly evaluated domains. Specifically, only one study (n=1, 0.92%) evaluated the user experience and satisfaction. The details are illustrated in Multimedia Appendix 4.

### Challenges and Opportunities With Wearable Devices

**Figure 5:**
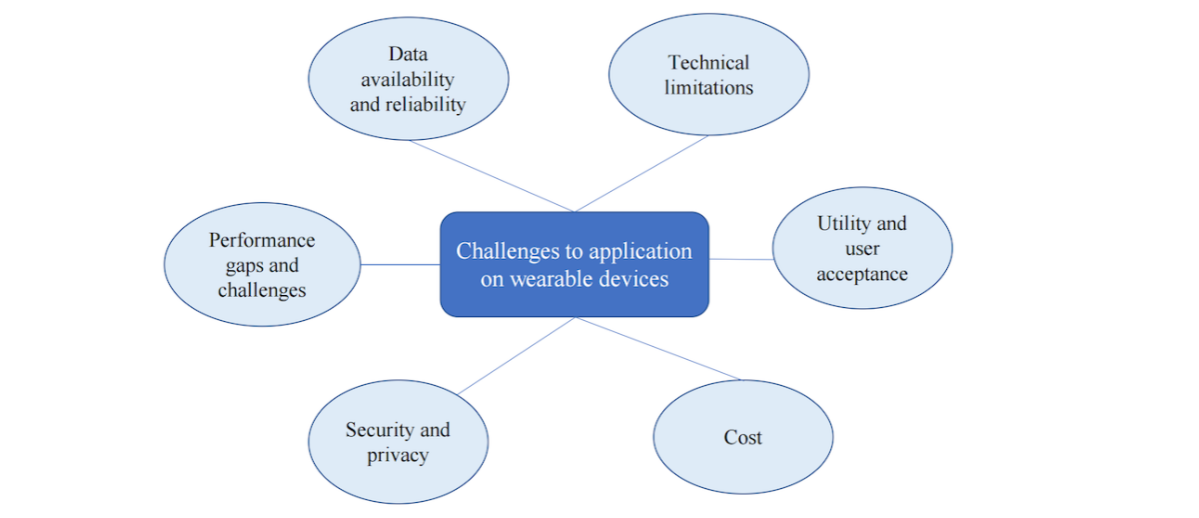
Categorization of challenges to wearable applications, showing proportions of the 6 categories.

There were several challenges and opportunities associated with wearable devices that were reported in studies or inductively emerged based on study descriptions (Table 4 and Figure 5). The opportunities presented by wearable devices are countered by multiple challenges, including data availability and reliability, technical limitations, utility and user acceptance, cost, security and privacy, performance gaps, and challenges.

**Table 4:** Categorization of challenges and opportunities with wearable devices.

Challenges		Opportunities
**Data availability and reliability**		
	Low quality of data acquired by sensors	Data validation and feedback： Ensure the quality and reliability of sensor data in wearable devices through data validation and user feedback. Optimizing algorithms: Improve data processing efficiency with more efficient algorithms. Enhance data quality through machine learning and data cleaning. Adding new sensors: Introduce higher precision sensors to improve data accuracy. Expand functionality with additional sensors for more physiological parameters. Common and local standards of data quality.
	Existing data samples lack representativeness and are relatively small	Expanding sample diversity and size: Use multicenter study designs to cover more geographic locations. Collaborate with multiple institutions to share datasets and expand sample size. In compliance with international data protection laws, data sources should be made accessible to researchers as far as possible.
**Technical limitation**		
	Interoperability of devices, short battery life, large device size, lack of personalized settings, and inadequate support	Engaging with older adults and seeking their input on the design and operation of these digital devices, understanding user needs, and achieving this goal.
**Utility and user acceptance**		
	Devices may not be available for independent use at home Users may have difficulty in learning new skills and high dependence on health care providers for health management	Technology dissemination and training： Develop effective training methods to master the use of technology, such as through simplified guides and increased use of visual tutorials.
	Devices may not cater to individual preferences, leading to a less satisfactory user experience	Involve users in the design and evaluation of technology products to obtain more direct and effective feedback. Improve the design of wearable devices and develop easy-to-understand and operational applications or interfaces.
**Cost**		
	High technological costs and ongoing support may bring higher costs	Rigorous validation from evidence-based practice, providing support, and lowering the cost for better financial sustainability. Assessing the cost-effectiveness of technology, facilitating broader adoption and application.
**Security and privacy**		
	Security risks include but are not limited to malicious hardware or software injections, denial-of-service attacks, and different routing and physical attacks	Homomorphic Encryption: Use homomorphic encryption to allow computations on encrypted data. Software Updates: Regularly update the firmware and software on wearable devices to patch known security vulnerabilities and improve overall security. Regulatory Compliance: Ensure compliance with relevant data protection regulations such as GDPRa, HIPAAb, and CCPAc.
**Performance gaps and challenges**		
	Lack of evaluations in real-world settings to adopt wearable devices for health care tasks	Large-scale clinical trials involving older adults to assess and validate their accuracy

^a^GDPR: General Data Protection Regulation.

^b^HIPAA: Health Insurance Portability and Accountability Act.

^c^CCPA: California Consumer Privacy Act.

## Discussion

### Principal Findings

Our study is the first to conduct a systematic scoping review and create an evidence map to provide an overview of the state of evidence regarding the development and evaluation of wearable devices. A total of 109 studies on wearable devices were included in our systematic review. This study provided a detailed mapping of existing wearable devices, outlining their basic characteristics, health targets, and underlying technologies, while also showing the evaluating results and identifying the challenges and opportunities associated with their use by older adults.

Mobility, fall-related incidents, and mental health monitoring are the primary focuses of wearable devices designed for older adults. Mobility-focused devices often include features such as step counting, gait analysis, and balance tracking to help users maintain their physical activity levels, promote healthy behaviors like exercise, and offer a wide range of applications in managing various diseases, such as high-risk older patients in the preoperative stage [126], hospitalized older people [127], patients with cancer [128], those with Parkinson disease [129], and those with COPD [130]. Faller classification in older adults can facilitate preventative care before a fall occurs. While mobility is prominent, mental health and cognition also remain important considerations for wearable technology targeting older adults. Early identification and monitoring of mental illnesses may be quite challenging. Contrary to the results of traditional patient self-reports, wearable devices could assess psychological states by continuously monitoring other indicators. Chan et al [131] used wrist-worn accelerometers to investigate whether gait biomarkers could predict the incidence of depressive episodes in older individuals and indeed found that these biomarkers can serve as predictive indicators. Toosizadeh et al [132] confirmed significant associations between upper-extremity speed, range of motion, and speed variability with both the Montreal Cognitive Assessment (MoCA) score and the gait performance within the dual-task condition. Vital signs monitoring is a major component of wearable devices, primarily including heart rate. The detection and diagnosis of atrial fibrillation (AF) are often challenging due to the asymptomatic and intermittent nature of AF. For patients who may be at risk of cardiac arrest, wearable devices can help monitor arrhythmias. The novel Necklace-ECG device is able to detect AF obtained with novel Necklace-ECG device [132], mobile device [133,134]. Santala et al [135] have demonstrated that an all-time wearable Necklace-ECG can detect and diagnose AF with high accuracy. In addition, there were measurements of physiological indicators, such as sleep calmness. Abbas et al [136] use devices that monitor daily steps, sleep duration, and quality, as well as activity levels, to distinguish physical frailty from daily routine. In summary, existing evidence on the broad implementation of wearable devices in clinical care highlights their benefits, which include enabling patient-centered care, enhancing the monitoring of physical activity, facilitating timely treatment adjustments, reducing the occurrence of unexpected events, and minimizing the need for unnecessary clinic visits.

Our systematic review explores how wearable devices are evaluated. Comparing sensor technologies with clinical scales and performance measures in a controlled laboratory and clinical setting is crucial for evaluating device effectiveness in specific tasks. The performance of the algorithm depends on several factors, including the dataset used, the type of classifier, the data acquisition device, and the ratio of training to testing data [137]. Performance testing of wearable devices typically evaluates key metrics such as accuracy (data and classification), reliability (stability and failure rates), precision and recall, sensitivity and specificity, and *F*_1_-score. Researchers have noticed this lack of user participation in single wearable real-world studies, and it has been identified as the main challenge in this field [138]. Although there has been an increasing trend in studies focusing on using wearable devices for predicting or quantifying the response to an intervention. In addition to effectiveness, only a very small number of studies have investigated the acceptability of the technology, user adherence, user experience, device cost, and safety. For example, Ward et al [130] reported on findings from a single-arm trial that an activity monitor technology could be able to support effective remote walking exercise prescription and participation during pulmonary rehabilitation in COPD. Preliminary findings from 3 studies [139-141], which assessed safety by reporting on related adverse events, indicated that the intervention was safe and well-accepted by the participants. Conducting a cost-benefit comparison between programs implemented in health care systems is challenging, and only one study explored the costs of home pedometer-assisted physical activity in patients with COPD [142]. The Model for Assessment of Telemedicine Applications (MATA) offers a structured framework for the multidisciplinary evaluation of telemedicine applications. This model assists decision makers in selecting the most efficient and cost-effective technologies, taking into account the specific characteristics of the application, safety, clinical efficacy, patient perspectives, and economic, organizational, sociocultural, ethical, and legal considerations [143]. Due to the limitations of laboratory studies in terms of their observation window (min to h) and the environment (generally safe, no obstacles) [144,145]. Researchers and developers can gain a deeper understanding of the real-world impact and effectiveness of wearable devices in health monitoring [146], ultimately leading to better-designed and more impactful solutions for end users. Wearable technology may be able to support effective remote walking.

There are many barriers to the full clinical adoption of digital monitoring, including technical limitations that hinder usability and satisfaction, as well as the lack of clear guidelines for wearable applications and data collection. First, the technical limitations of wearable devices, including interoperability issues, short battery life, large device size, lack of personalized settings, and inadequate support, restrict their application scenarios and may lead to a less satisfactory user experience by not catering to individual preferences [147,148]. Given the limitations observed in the structural design and practical applications of wearable technology, we can undertake several optimizations to improve their effectiveness in clinical practice. Second, our review demonstrated that there is substantial heterogeneity among the wearable sensor technologies used for the assessment of older adults. A large proportion of the devices, their potential benefits, and utility are still far from implementation in clinical routine [149]. Besides, future research should also focus on identifying factors that could enhance the effectiveness of wearable technologies and promote their acceptance and adherence in the longer term. Such factors include the digital technology types (eg, user-friendly design and personalized settings) and the nondigital elements in digital health interventions (eg, motivation and engagement) [25]. Third, wearable devices face major challenges including inadequate data availability and reliability, as well as privacy protection concerns [150]. Food and Drug Administration [151] and the European Medicines Agency [152] have developed guidelines for the use, validation, and data reporting of wearable sensor technology in clinical trials. However, these guidelines are not yet widely known, and journals should recommend and even require their adoption to disseminate these best practices [153,154]. Last, given the complexity of human responses and the variability among individuals, data collection can be quite challenging [155]. However, the data collection process is often both costly and time-consuming, leading most academic studies to rely on relatively limited datasets. To address this, it is advisable to gather as much data as possible from participants in order to develop robust algorithms for data cleansing and to construct more accurate and broadly applicable models. Adopting a transparent and reproducible approach to data collection, training, and model evaluation is highly recommended to bolster confidence in research outcomes and to facilitate the effective accumulation of research efforts [156].

Despite many barriers to their application, wearable devices have brought huge benefits to the aging global population. In addition, their impact on health care is profound as the younger, technologically literate generation ages into adulthood. On the one hand, the potential to significantly boost the technological literacy of future older populations becomes more apparent. Currently, older adults may have faced a double exclusion due to a lack of digital literacy and social interaction who rely on health monitoring technologies for personalized medical guidance [157]. In contrast, future cohorts of older adults, being more adept with data and platforms, may actively better understand the data and recommendations provided by the devices, leading to more active participation in remote health education programs. On the other hand, the dynamic and continuous accumulation of health data contrasts sharply with traditional, static, and episodic medical history records. This shift has the potential to revolutionize the entire health care system, moving health care from a “diagnosis-treatment model” toward a “prediction-prevention model.” For instance, existing research indicates that early signs of cognitive function may be identified through sleep data [158]. Consequently, future medical services may place greater emphasis on the prediction and prevention of disease, potentially reducing the need for treatment approaches.

### Evidence Gaps and Ideas for Future Research

Furthermore, improvements are needed for (1) research should include a broader and more diverse range of older adults to ensure that the findings are applicable to various demographic groups, which includes considering differences in age, gender, socioeconomic status, and cultural backgrounds, (2) understanding the underlying physiological processes that these technologies monitor, and influence can lead to more effective and targeted interventions. This includes studying how sensors and algorithms interact with the body’s biological signals to provide accurate and reliable data, (3) research should extend beyond laboratory settings to include various clinical and care environments. This will help identify the practical challenges and benefits of using wearable technologies in real-world scenarios, such as hospitals, nursing homes, and home care settings, (4) outcome evaluations are necessary to identify the factors that can enhance the health benefits of digital technologies. This includes assessing both short- and long-term outcomes, with a focus on health technology assessments, including the effectiveness, patient safety, and impact on health care costs, (5) Technologically literate older adults may be more proactive in managing their health, leveraging wearables for continuous monitoring of vital signs, chronic conditions, and mental health. Adherence could be further enhanced with the provision of additional cognitive aids, such as detailed documentation of study procedures and comprehensive user manuals, as well as by allowing sufficient time for users to establish wearing routines [11], (6) by integrating quantitative and qualitative insights from participants, we can further refine wearable device technology. The research about how to simplify interfaces and operations to make them more intuitive for older adults, while exploring ways to tailor devices based on individual health conditions, lifestyle, and preferences to provide personalized health management and reminder services.

### Limitations

First, not all research involving wearable devices was identified by our search. For example, some company products may not appear in databases. Due to practical constraints, we included only studies published in the English language. Consequently, it is likely that we missed some studies published in other languages, potentially leading to an incomplete and potentially biased set of results. Second, we reported on the challenges and opportunities of wearable devices from the included studies. While these insights may be helpful, they are not objective measures. Last, the categories of research and the objectives for using wearable devices introduced in our study may overlap, as separation and classification are artificial.

### Conclusion

We see a growing uptake of wearable technology in health research with a notable trend toward its use in older adults. In the future, a broader and more diverse range of studies should be designed to investigate which types of wearable devices are most beneficial for older adults. In addition, it is important to explore the potential of emerging innovations in enhancing health outcomes. Focusing on optimizing wearable device technology to improve usability, and effectiveness is also crucial. Outcome evaluations are needed to identify factors that could enhance the health benefits of these technologies and to measure their real-world impact. This review can help nonmedical professionals and policy makers visualize and better understand wearable technology in future studies.

## Appendix

Multimedia Appendix 1 PRISMA-ScR checklist.

Multimedia Appendix 2 Search strategy.

Multimedia Appendix 3 Extracted data form.

Multimedia Appendix 4 The characteristics of the studies.

## Abbreviations

ACM: Association for Computing Machinery
AF: atrial fibrillation
COPD: chronic obstructive pulmonary disease
MATA: Model for Assessment of Telemedicine Applications
MeSH: Medical Subject Headings
MoCA: Montreal Cognitive Assessment
PCC: Population, Concept, and Context
PRISMA-ScR: Preferred Reporting Items for Systematic Reviews and Meta-Analyses extension for Scoping Reviews

## References

[1] Yetisen AK, Martinez-Hurtado JL, Ünal B, Khademhosseini A, Butt H (2018). Wearables in medicine. Adv Mater.

[2] Metcalf D, Milliard STJ, Gomez M, Schwartz M (2016). Wearables and the internet of things for health: wearable, interconnected devices promise more efficient and comprehensive health care. IEEE Pulse.

[3] Jo A, Coronel BD, Coakes CE, Mainous AG (2019). Is there a benefit to patients using wearable devices such as fitbit or health apps on mobiles? A systematic review. Am J Med.

[4] Sequeira L, Perrotta S, LaGrassa J, Merikangas K, Kreindler D, Kundur D, Courtney D, Szatmari P, Battaglia M, Strauss J (2020). Mobile and wearable technology for monitoring depressive symptoms in children and adolescents: a scoping review. J Affect Disord.

[5] Zhang W, Xiong K, Zhu C, Evans R, Zhou L, Podrini C (2024). Promoting child and adolescent health through wearable technology: a systematic review. Digit Health.

[6] Takei K, Honda W, Harada S, Arie T, Akita S (2015). Toward flexible and wearable human-interactive health-monitoring devices. Adv Healthc Mater.

[7] Lu L, Zhang J, Xie Y, Gao F, Xu S, Wu X, Ye Z (2020). Wearable health devices in health care: narrative systematic review. JMIR Mhealth Uhealth.

[8] Lewczak Z, Mitchell M (2024). Wearable technology and chronic illness: balancing justice and care ethics. Cureus.

[9] Matsuura H (2024). Further acceleration in fertility decline in 2023: deviation of recently published provisional fertility estimates in selected OECD countries from those in the 2022 revision of the world population prospects. Biodemography Soc Biol.

[10] Beard JR, Bloom DE (2015). Towards a comprehensive public health response to population ageing. Lancet.

[11] Guu TW, Muurling M, Khan Z, Kalafatis C, Aarsland D, Ffytche D, Brem AK (2023). Wearable devices: underrepresentation in the ageing society. Lancet Digit Health.

[12] Fuller D, Colwell E, Low J, Orychock K, Tobin MA, Simango B, Buote R, Van Heerden D, Luan H, Cullen K, Slade L, Taylor NGA (2020). Reliability and validity of commercially available wearable devices for measuring steps, energy expenditure, and heart rate: systematic review. JMIR Mhealth Uhealth.

[13] Helmer P, Hottenrott S, Rodemers P, Leppich R, Helwich M, Pryss R, Kranke P, Meybohm P, Winkler BE, Sammeth M (2022). Accuracy and systematic biases of heart rate measurements by consumer-grade fitness trackers in postoperative Patients: Prospective clinical trial. J Med Internet Res.

[14] Toly VB, Fiala M, Cohen S (2023). Postal delivery of sleep monitoring devices: research implications. Clin Nurs Res.

[15] Sabry F, Eltaras T, Labda W, Alzoubi K, Malluhi Q (2022). Machine learning for healthcare wearable devices: The big picture. J Healthc Eng.

[16] Hua A, Quicksall Z, Di C, Motl R, LaCroix AZ, Schatz B, Buchner DM (2018). Accelerometer-based predictive models of fall risk in older women: a pilot study. NPJ Digit Med.

[17] Zhang J, Li Z, Liu Y, Li J, Qiu H, Li M, Hou G, Zhou Z (2024). An effective deep learning framework for fall detection: model development and study design. J Med Internet Res.

[18] Rykov YG, Patterson MD, Gangwar BA, Jabar SB, Leonardo J, Ng KP, Kandiah N (2024). Predicting cognitive scores from wearable-based digital physiological features using machine learning: data from a clinical trial in mild cognitive impairment. BMC Med.

[19] Sakal C, Li T, Li J, Li X (2024). Predicting poor performance on cognitive tests among older adults using wearable device data and machine learning: a feasibility study. NPJ Aging.

[20] Slemenšek J, Fister I, Geršak J, Bratina B, van Midden VM, Pirtošek Z, Šafarič R (2023). Human gait activity recognition machine learning methods. Sensors (Basel).

[21] Khan Y, Ostfeld AE, Lochner CM, Pierre A, Arias AC (2016). Monitoring of vital signs with flexible and wearable medical devices. Adv Mater.

[22] Chen Q, Sheng N (2024). A novel health monitoring system for vital signs using IoT. Sci Rep.

[23] Chen S, Qi J, Fan S, Qiao Z, Yeo JC, Lim CT (2021). Flexible wearable sensors for cardiovascular health monitoring. Adv Healthc Mater.

[24] Canali S, Schiaffonati V, Aliverti A (2022). Challenges and recommendations for wearable devices in digital health: data quality, interoperability, health equity, fairness. PLOS Digit Health.

[25] De Santis KK, Mergenthal L, Christianson L, Busskamp A, Vonstein C, Zeeb H (2023). Digital technologies for health promotion and disease prevention in older people: Scoping review. J Med Internet Res.

[26] Moore K, O'Shea E, Kenny L, Barton J, Tedesco S, Sica M, Crowe C, Alamäki A, Condell J, Nordström A, Timmons S (2021). Older adults' experiences with using wearable devices: qualitative systematic review and meta-synthesis. JMIR Mhealth Uhealth.

[27] Chen C, Ding S, Wang J (2023). Digital health for aging populations. Nat Med.

[28] Sandic Spaho R, Uhrenfeldt L, Fotis T, Kymre IG (2023). Wearable devices in palliative care for people 65 years and older: a scoping review. Digit Health.

[29] Hughes A, Shandhi MMH, Master H, Dunn J, Brittain E (2023). Wearable devices in cardiovascular medicine. Circ Res.

[30] Ahmed A, Aziz S, Alzubaidi M, Schneider J, Irshaidat S, Abu Serhan H, Abd-Alrazaq AA, Solaiman B, Househ M (2023). Comput Methods Programs Biomed Update.

[31] Gagnon MP, Ouellet S, Attisso E, Supper W, Amil S, Rhéaume C, Paquette J, Chabot C, Laferrière MC, Sasseville M (2024). Wearable devices for supporting chronic disease self-management: Scoping review. Interact J Med Res.

[32] Tricco AC, Antony J, Zarin W, Strifler L, Ghassemi M, Ivory J, Perrier L, Hutton B, Moher D, Straus SE (2015). A scoping review of rapid review methods. BMC Med.

[33] Miake-Lye IM, Hempel S, Shanman R, Shekelle PG (2016). What is an evidence map? A systematic review of published evidence maps and their definitions, methods, and products. Syst Rev.

[34] Arksey H, O'Malley L (2005). Scoping studies: towards a methodological framework. Int J Soc Res Methodol.

[35] Tricco AC, Lillie E, Zarin W, O'Brien KK, Colquhoun H, Levac D, Moher D, Peters MDJ, Horsley T, Weeks L, Hempel S, Akl EA, Chang C, McGowan J, Stewart L, Hartling L, Aldcroft A, Wilson MG, Garritty C, Lewin S, Godfrey CM, Macdonald MT, Langlois EV, Soares-Weiser K, Moriarty J, Clifford T, Tunçalp Ö, Straus SE (2018). PRISMA Extension for scoping reviews (PRISMA-ScR): checklist and explanation. Ann Intern Med.

[36] Levac D, Colquhoun H, O'Brien KK (2010). Scoping studies: advancing the methodology. Implement Sci.

[37] Choi J, Lee S, Kim S, Kim D, Kim H (2022). Depressed mood prediction of elderly people with a wearable band. Sensors (Basel).

[38] Kim H, Lee SH, Lee SE, Hong S, Kang HJ, Kim N (2019). Depression prediction by using ecological momentary assessment, actiwatch data, and machine learning: Observational study on older adults living alone. JMIR Mhealth Uhealth.

[39] Nath RK, Thapliyal H (2021). Machine learning-based anxiety detection in older adults using wristband sensors and context feature. SN Comput Sci.

[40] Alharbi M, Bauman A, Neubeck L, Gallagher R (2016). Validation of fitbit-flex as a measure of free-living physical activity in a community-based phase III cardiac rehabilitation population. Eur J Prev Cardiol.

[41] Burton E, Hill KD, Lautenschlager NT, Thøgersen-Ntoumani C, Lewin G, Boyle E, Howie E (2018). Reliability and validity of two fitness tracker devices in the laboratory and home environment for older community-dwelling people. BMC Geriatr.

[42] Compagnat M, Mandigout S, Chaparro D, Daviet JC, Salle JY (2018). Validity of the actigraph GT3x and influence of the sensor positioning for the assessment of active energy expenditure during four activities of daily living in stroke subjects. Clin Rehabil.

[43] Lamont RM, Daniel HL, Payne CL, Brauer SG (2018). Accuracy of wearable physical activity trackers in people with parkinson's disease. Gait Posture.

[44] Magistro D, Brustio PR, Ivaldi M, Esliger DW, Zecca M, Rainoldi A, Boccia G (2018). Validation of the ADAMO care watch for step counting in older adults. PLoS One.

[45] Mandigout S, Lacroix J, Ferry B, Vuillerme N, Compagnat M, Daviet JC (2017). Can energy expenditure be accurately assessed using accelerometry-based wearable motion detectors for physical activity monitoring in post-stroke patients in the subacute phase?. Eur J Prev Cardiol.

[46] Cooper C, Gross A, Brinkman C, Pope R, Allen K, Hastings S, Bogen BE, Goode AP (2018). The impact of wearable motion sensing technology on physical activity in older adults. Exp Gerontol.

[47] Larsen RT, Christensen J, Juhl CB, Andersen HB, Langberg H (2019). Physical activity monitors to enhance amount of physical activity in older adults - a systematic review and meta-analysis. Eur Rev Aging Phys Act.

[48] Liu JYW, Kor PPK, Chan CPY, Kwan RYC, Cheung DSK (2020). The effectiveness of a wearable activity tracker (WAT)-based intervention to improve physical activity levels in sedentary older adults: a systematic review and meta-analysis. Arch Gerontol Geriatr.

[49] S Oliveira J, Sherrington C, R Y Zheng E, Franco MR, Tiedemann A (2020). Effect of interventions using physical activity trackers on physical activity in people aged 60 years and over: a systematic review and meta-analysis. Br J Sports Med.

[50] Grimes L, Outtrim JG, Griffin SJ, Ercole A (2019). Accelerometery as a measure of modifiable physical activity in high-risk elderly preoperative patients: a prospective observational pilot study. BMJ Open.

[51] Jonker LT, Hendriks S, Lahr MM, van Munster BC, de Bock GH, van Leeuwen BL (2020). Postoperative recovery of accelerometer-based physical activity in older cancer patients. Eur J Surg Oncol.

[52] Kabbach EZ, Mazzuco A, Borghi-Silva A, Cabiddu R, Agnoleto AG, Barbosa JF, de Carvalho Junior LCS, Mendes RG (2017). Increased parasympathetic cardiac modulation in patients with acute exacerbation of COPD: how should we interpret it?. Int J Chron Obstruct Pulmon Dis.

[53] Kimura N, Aso Y, Yabuuchi K, Ishibashi M, Hori D, Sasaki Y, Nakamichi A, Uesugi S, Jikumaru M, Sumi K, Eguchi A, Obara H, Kakuma T, Matsubara E (2020). Association of modifiable lifestyle factors with cortical amyloid burden and cerebral glucose metabolism in older adults with mild cognitive impairment. JAMA Netw Open.

[54] Yang Y, Hirdes JP, Dubin JA, Lee J (2019). Fall risk classification in community-dwelling older adults using a smart wrist-worn device and the resident assessment instrument-home care: prospective observational study. JMIR Aging.

[55] Lin LF, Lin YJ, Lin ZH, Chuang LY, Hsu WC, Lin YH (2018). Feasibility and efficacy of wearable devices for upper limb rehabilitation in patients with chronic stroke: a randomized controlled pilot study. Eur J Phys Rehabil Med.

[56] Zhang S, Xian H, Chen Y, Liao Y, Zhang N, Guo X, Yang M, Wu J (2021). The auxiliary diagnostic value of a novel wearable electrocardiogram-recording system for arrhythmia detection: diagnostic trial. Front Med (Lausanne).

[57] Heo NJ, Rhee SY, Waalen J, Steinhubl S (2020). Chronic kidney disease and undiagnosed atrial fibrillation in individuals with diabetes. Cardiovasc Diabetol.

[58] Reverberi C, Rabia G, De Rosa F, Bosi D, Botti A, Benatti G (2019). The RITMIA™ Smartphone app for automated detection of atrial fibrillation: Accuracy in consecutive patients undergoing elective electrical cardioversion. Biomed Res Int.

[59] Proesmans T, Mortelmans C, Van Haelst R, Verbrugge F, Vandervoort P, Vaes B (2019). Mobile phone-based use of the photoplethysmography technique to detect atrial fibrillation in primary care: diagnostic accuracy study of the FibriCheck app. JMIR Mhealth Uhealth.

[60] Lown M, Yue AM, Shah BN, Corbett SJ, Lewith G, Stuart B, Garrard J, Brown M, Little P, Moore M (2018). Screening for atrial fibrillation using economical and accurate technology (from the SAFETY study). Am J Cardiol.

[61] Baston C, Mancini M, Schoneburg B, Horak F, Rocchi L (2014). Postural strategies assessed with inertial sensors in healthy and parkinsonian subjects. Gait Posture.

[62] Fazio P, Granieri G, Casetta I, Cesnik E, Mazzacane S, Caliandro P, Pedrielli F, Granieri E (2013). Gait measures with a triaxial accelerometer among patients with neurological impairment. Neurol Sci.

[63] Gago MF, Fernandes V, Ferreira J, Silva H, Rodrigues ML, Rocha L, Bicho E, Sousa N (2015). The effect of levodopa on postural stability evaluated by wearable inertial measurement units for idiopathic and vascular parkinson's disease. Gait Posture.

[64] Hasmann SE, Berg D, Hobert MA, Weiss D, Lindemann U, Streffer J, Liepelt-Scarfone I, Maetzler W (2014). Instrumented functional reach test differentiates individuals at high risk for parkinson's disease from controls. Front Aging Neurosci.

[65] Herman T, Weiss A, Brozgol M, Giladi N, Hausdorff JM (2014). Gait and balance in Parkinson's disease subtypes: objective measures and classification considerations. J Neurol.

[66] Armstrong M, Hume E, McNeillie L, Chambers F, Wakenshaw L, Burns G, Marshall KH, Vogiatzis I (2021). Behavioural modification interventions alongside pulmonary rehabilitation improve COPD patients' experiences of physical activity. Respir Med.

[67] Brickwood KJ, Ahuja KDK, Watson G, O'Brien JA, Williams AD (2021). Effects of activity tracker use with health professional support or telephone counseling on maintenance of physical activity and health outcomes in older adults: randomized controlled trial. JMIR Mhealth Uhealth.

[68] Muellmann S, Buck C, Voelcker-Rehage C, Bragina I, Lippke S, Meyer J, Peters M, Pischke CR (2019). Effects of two web-based interventions promoting physical activity among older adults compared to a delayed intervention control group in Northwestern Germany: Results of the PROMOTE community-based intervention trial. Prev Med Rep.

[69] Martínez-Ramírez A, Lecumberri P, Gómez M, Rodriguez-Mañas L, García FJ, Izquierdo M (2011). Frailty assessment based on wavelet analysis during quiet standing balance test. J Biomech.

[70] Theou O, Jakobi JM, Vandervoort AA, Jones GR (2012). A comparison of physical activity (PA) assessment tools across levels of frailty. Arch Gerontol Geriatr.

[71] Galán-Mercant A, Cuesta-Vargas AI (2013). Differences in trunk accelerometry between frail and nonfrail elderly persons in sit-to-stand and stand-to-sit transitions based on a mobile inertial sensor. JMIR Mhealth Uhealth.

[72] Greene BR, Doheny EP, O'Halloran A, Anne Kenny R (2014). Frailty status can be accurately assessed using inertial sensors and the TUG test. Age Ageing.

[73] Toosizadeh N, Mohler J, Wendel C, Najafi B (2015). Influences of frailty syndrome on open-loop and closed-loop postural control strategy. Gerontology.

[74] Toosizadeh N, Mohler J, Najafi B (2015). Assessing upper extremity motion: an innovative method to identify frailty. J Am Geriatr Soc.

[75] Parvaneh S, Mohler J, Toosizadeh N, Grewal GS, Najafi B (2017). Postural transitions during activities of daily living could identify frailty status: application of wearable technology to identify frailty during unsupervised condition. Gerontology.

[76] Schwenk M, Mohler J, Wendel C, D'Huyvetter K, Fain M, Taylor-Piliae R, Najafi B (2015). Wearable sensor-based in-home assessment of gait, balance, and physical activity for discrimination of frailty status: baseline results of the Arizona frailty cohort study. Gerontology.

[77] Jansen FM, Prins RG, Etman A, van der Ploeg HP, de Vries SI, van Lenthe FJ, Pierik FH (2015). Physical activity in non-frail and frail older adults. PLoS One.

[78] Martínez-Ramírez A, Martinikorena I, Gómez M, Lecumberri P, Millor N, Rodríguez-Mañas L, García García FJ, Izquierdo M (2015). Frailty assessment based on trunk kinematic parameters during walking. J Neuroeng Rehabil.

[79] Huisingh-Scheetz M, Wroblewski K, Kocherginsky M, Huang E, Dale W, Waite L, Schumm LP (2018). The relationship between physical activity and frailty among U.S. older adults based on hourly accelerometry data. J Gerontol A Biol Sci Med Sci.

[80] Mulasso A, Brustio PR, Rainoldi A, Zia G, Feletti L, N'dja A, Del Signore S, Poggiogalle E, Luisi F, Donini LM (2019). A comparison between an ICT tool and a traditional physical measure for frailty evaluation in older adults. BMC Geriatr.

[81] Zhou H, Razjouyan J, Halder D, Naik AD, Kunik ME, Najafi B (2019). Instrumented trail-making task: application of wearable sensor to determine physical frailty phenotypes. Gerontology.

[82] Apsega A, Petrauskas L, Alekna V, Daunoraviciene K, Sevcenko V, Mastaviciute A, Vitkus D, Tamulaitiene M, Griskevicius J (2020). Wearable sensors technology as a tool for discriminating frailty levels during instrumented gait analysis. Appl Sci.

[83] Casilari E, Oviedo-Jiménez MA (2015). Automatic fall detection system based on the combined use of a smartphone and a smartwatch. PLoS One.

[84] Rakhman AZK, Nugrohoi LE, Widyawani D (2014). u-F ASt: Ubiquitous fall detection and alert system for elderly people in smart home environment.

[85] Shahzad A, Ko S, Lee S, Lee J, Kim K (2017). Quantitative assessment of balance impairment for fall-risk estimation using wearable triaxial accelerometer. IEEE Sensors J.

[86] Howcroft J, Kofman J, Lemaire ED (2017). Prospective fall-risk prediction models for older adults based on wearable sensors. IEEE Trans Neural Syst Rehabil Eng.

[87] Greene BR, Redmond SJ, Caulfield B (2017). Fall risk assessment through automatic combination of clinical fall risk factors and body-worn sensor data. IEEE J Biomed Health Inform.

[88] Drover D, Howcroft J, Kofman J, Lemaire ED (2017). Faller classification in older adults using wearable sensors based on turn and straight-walking accelerometer-based features. Sensors (Basel).

[89] Howcroft J, Lemaire ED, Kofman J (2018). Prospective elderly fall prediction by older-adult fall-risk modeling with feature selection. Biomed Signal Process Control.

[90] Ghahramani M, Stirling D, Naghdy F, Naghdy G, Potter J (2019). Body postural sway analysis in older people with different fall histories. Med Biol Eng Comput.

[91] Buisseret F, Catinus L, Grenard R, Jojczyk L, Fievez D, Barvaux V, Dierick F (2020). Timed up and go and six-minute walking tests with wearable inertial sensor: one step further for the prediction of the risk of fall in elderly nursing home people. Sensors (Basel).

[92] Yu L, Zhao Y, Wang H, Sun TL, Murphy TE, Tsui KL (2021). Assessing elderly's functional balance and mobility via analyzing data from waist-mounted tri-axial wearable accelerometers in timed up and go tests. BMC Med Inform Decis Mak.

[93] Lockhart TE, Soangra R, Yoon H, Wu T, Frames CW, Weaver R, Roberto KA (2021). Prediction of fall risk among community-dwelling older adults using a wearable system. Sci Rep.

[94] Choi J, Parker SM, Knarr BA, Gwon Y, Youn JH (2021). Wearable sensor-based prediction model of timed up and go test in older adults. Sensors (Basel).

[95] Wu S, Ou J, Shu L, Hu G, Song Z, Xu X, Chen Z (2022). MhNet: Multi-scale spatio-temporal hierarchical network for real-time wearable fall risk assessment of the elderly. Comput Biol Med.

[96] Tunca C, Salur G, Ersoy C (2020). Deep learning for fall risk assessment with inertial sensors: utilizing domain knowledge in spatio-temporal gait parameters. IEEE J Biomed Health Inform.

[97] Duscha BD, Piner LW, Patel MP, Craig KP, Brady M, McGarrah RW, Chen C, Kraus WE (2018). Effects of a 12-week mHealth program on peak VO and physical activity patterns after completing cardiac rehabilitation: a randomized controlled trial. Am Heart J.

[98] Henriksen A, Haugen Mikalsen M, Woldaregay AZ, Muzny M, Hartvigsen G, Hopstock LA, Grimsgaard S (2018). Using fitness trackers and smartwatches to measure physical activity in research: analysis of consumer wrist-worn wearables. J Med Internet Res.

[99] Thompson WG, Kuhle CL, Koepp GA, McCrady-Spitzer SK, Levine JA (2014). "Go4Life" exercise counseling, accelerometer feedback, and activity levels in older people. Arch Gerontol Geriatr.

[100] Kirkendall ES, McCraw J, Ganesh S, Lang S, Mariotti M, Evered M, Ghoreyshi A, Williamson J, Zamora Z (2022). Feasibility, acceptability, and performance of a continuous temperature monitor in older adults and staff in congregate-living facilities. J Am Med Dir Assoc.

[101] Park HK, Jung J, Lee DW, Shin HC, Lee HJ, Lee WH (2022). A wearable electromyography-controlled functional electrical stimulation system improves balance, gait function, and symmetry in older adults. Technol Health Care.

[102] Chang CH, Lien WC, Chiu TP, Yang TH, Wei CC, Kuo YL, Yeh CH, Liu B, Chen PJ, Lin YC (2023). A novel smart somatosensory wearable assistive device for older adults' home rehabilitation during the COVID-19 pandemic. Front Public Health.

[103] Seok JW, Kwon YJ, Lee H (2022). Feasibility and efficacy of TouchCare system using application for older adults living alone: a pilot pre-experimental study. BMC Geriatr.

[104] Lee SH, Kim J, Lim B, Lee HJ, Kim YH (2023). Exercise with a wearable hip-assist robot improved physical function and walking efficiency in older adults. Sci Rep.

[105] Shin JH, Byeon N, Yu H, Yun G, Kim H, Lim S, Kim D, Lee HJ, Lee WH (2023). Effect of 4-weeks exercise program using wearable hip-assist robot (EX1) in older adults: one group pre- and post- test. BMC Geriatr.

[106] Kim HJ, Kim H, Park J, Oh B, Kim SC (2022). Recognition of gait patterns in older adults using wearable smartwatch devices: observational study. J Med Internet Res.

[107] Garcia-Moreno FM, Bermudez-Edo M, Rodríguez-García E, Pérez-Mármol JM, Garrido JL, Rodríguez-Fórtiz MJ (2022). A machine learning approach for semi-automatic assessment of IADL dependence in older adults with wearable sensors. Int J Med Inform.

[108] Wong AKC, Bayuo J, Su JJ, Chow KKS, Wong SM, Wong BP, Lee AYL, Wong FKY (2024). Exploring the experiences of community-dwelling older adults on using wearable monitoring devices with regular support from community health workers, nurses, and social workers: qualitative descriptive study. J Med Internet Res.

[109] Zadka A, Rabin N, Gazit E, Mirelman A, Nieuwboer A, Rochester L, Del Din S, Pelosin E, Avanzino L, Bloem BR, Della Croce U, Cereatti A, Hausdorff JM (2024). A wearable sensor and machine learning estimate step length in older adults and patients with neurological disorders. NPJ Digit Med.

[110] Koivunen K, Löppönen A, Palmberg L, Rantalainen T, Rantanen T, Karavirta L (2023). Autonomic nervous system and postural control regulation during orthostatic test as putative markers of physical resilience among community-dwelling older adults. Exp Gerontol.

[111] Kerstiens S, Bender EM, Rizzo MG, Landi A, Gleason LJ, Huisingh-Scheetz M, Rubin D, Ferguson M, Madariaga MLL (2023). Technology-assisted behavioral intervention to encourage prehabilitation in frail older adults undergoing surgery: Development and design of the BeFitMe™ apple watch app. Digit Health.

[112] Zhang Y, Wang X, Han P, Verschueren S, Chen W, Vanrumste B (2022). Can wearable devices and machine learning techniques be used for recognizing and segmenting modified physical performance test items?. IEEE Trans Neural Syst Rehabil Eng.

[113] Muñoz Esquivel K, Gillespie J, Kelly D, Condell J, Davies R, McHugh C, Duffy W, Nevala E, Alamäki A, Jalovaara J, Tedesco S, Barton J, Timmons S, Nordström A (2023). Factors influencing continued wearable device use in older adult populations: quantitative study. JMIR Aging.

[114] Oliveira LKR, Marques AP, Igarashi Y, Andrade KFA, Souza GS, Callegari B (2023). Wearable-based assessment of anticipatory postural adjustments during step initiation in patients with knee osteoarthritis. PLoS One.

[115] Lee WJ, Peng LN, Lin MH, Loh CH, Chen LK (2021). Active wearable device utilization improved physical performance and IGF-1 among community-dwelling middle-aged and older adults: a 12-month prospective cohort study. Aging (Albany NY).

[116] Mancioppi G, Rovini E, Fiorini L, Zeghari R, Gros A, Manera V, Robert P, Cavallo F (2023). Mild cognitive impairment identification based on motor and cognitive dual-task pooled indices. PLoS One.

[117] Camp N, Johnston J, Lewis MGC, Zecca M, Di Nuovo A, Hunter K, Magistro D (2022). Perceptions of in-home monitoring technology for activities of daily living: semistructured interview study with community-dwelling older adults. JMIR Aging.

[118] Park J, Lee HJ, Park JS, Kim CH, Jung WJ, Won S, Bae JB, Han JW, Kim KW (2023). Development of a gait feature-based model for classifying cognitive disorders using a single wearable inertial sensor. Neurology.

[119] Han D, Ding EY, Cho C, Jung H, Dickson EL, Mohagheghian F, Peitzsch AG, DiMezza D, Tran KV, McManus DD, Chon KH (2023). A smartwatch system for continuous monitoring of atrial fibrillation in older adults after stroke or transient ischemic attack: application design study. JMIR Cardio.

[120] Mughal F, Raffe W, Stubbs P, Kneebone I, Garcia J (2022). Fitbits for monitoring depressive symptoms in older aged persons: qualitative feasibility study. JMIR Form Res.

[121] Ram M, Baltzopoulos V, Shaw A, Maganaris CN, Cullen J, O'Brien T, Kot P (2023). A novel smart shoe instrumented with sensors for quantifying foot placement and clearance during stair negotiation. Sensors (Basel).

[122] Howcroft J, Lemaire ED, Kofman J (2016). Wearable-sensor-based classification models of faller status in older adults. PLoS One.

[123] Wang J, Carroll D, Peck M, Myneni S, Gong Y (2016). Mobile and wearable technology needs for aging in place: perspectives from older adults and their caregivers and providers. Stud Health Technol Inform.

[124] Joseph B, Toosizadeh N, Orouji Jokar T, Heusser MR, Mohler J, Najafi B (2017). Upper-extremity function predicts adverse health outcomes among older adults hospitalized for ground-level falls. Gerontology.

[125] Batsis JA, Naslund JA, Gill LE, Masutani RK, Agarwal N, Bartels SJ (2016). Use of a wearable activity device in rural older obese adults:a pilot study. Gerontol Geriatr Med.

[126] Chang YT (2020). Physical activity and cognitive function in mild cognitive impairment. ASN Neuro.

[127] Lim SER, Dodds R, Bacon D, Sayer AA, Roberts HC (2018). Physical activity among hospitalised older people: insights from upper and lower limb accelerometry. Aging Clin Exp Res.

[128] Jonker LT, Hendriks S, Lahr MM, van Munster BC, de Bock GH, van Leeuwen BL (2020). Postoperative recovery of accelerometer-based physical activity in older cancer patients. Eur J Surg Oncol.

[129] Pradhan S, Kelly VE (2019). Quantifying physical activity in early parkinson disease using a commercial activity monitor. Parkinsonism Relat Disord.

[130] Ward S, Orme M, Zatloukal J, Singh S (2021). Adherence to walking exercise prescription during pulmonary rehabilitation in COPD with a commercial activity monitor: a feasibility trial. BMC Pulm Med.

[131] Chan LLY, Brodie MA, Lord SR (2023). Prediction of incident depression in middle-aged and older adults using digital gait biomarkers extracted from large-scale wrist sensor data. J Am Med Dir Assoc.

[132] Toosizadeh N, Najafi B, Reiman EM, Mager RM, Veldhuizen JK, O'Connor K, Zamrini E, Mohler J (2016). Upper-extremity dual-task function: an innovative method to assess cognitive impairment in older adults. Front Aging Neurosci.

[133] Chan NY, Choy CC, Chan CK, Siu CW (2018). Effectiveness of a nongovernmental organization-led large-scale community atrial fibrillation screening program using the smartphone electrocardiogram: an observational cohort study. Heart Rhythm.

[134] Fabritz L, Connolly D, Czarnecki E, Dudek D, Zlahoda-Huzior A, Guasch E, Haase D, Huebner T, Jolly K, Kirchhof P, Schotten U, Zapf A, Schnabel RB (2022). Remote design of a smartphone and wearable detected atrial arrhythmia in older adults case finding study: Smart in OAC - AFNET 9. Front Cardiovasc Med.

[135] Santala OE, Lipponen JA, Jäntti H, Rissanen TT, Halonen J, Kolk I, Pohjantähti-Maaroos H, Tarvainen MP, Väliaho ES, Hartikainen J, Martikainen T (2021). Necklace-embedded electrocardiogram for the detection and diagnosis of atrial fibrillation. Clin Cardiol.

[136] Abbas M, Saleh M, Somme D, Le Bouquin Jeannès R (2023). Data-driven systems to detect physical weakening from daily routine: a pilot study on elderly over 80 years old. PLoS One.

[137] Dietterich TG (1998). Approximate statistical tests for comparing supervised classification learning algorithms. Neural Comput.

[138] Sun Y, Zhou J, Ji M, Pei L, Wang Z (2023). Development and evaluation of health recommender systems: Systematic scoping review and evidence mapping. J Med Internet Res.

[139] Lyons EJ, Swartz MC, Lewis ZH, Martinez E, Jennings K (2017). Feasibility and acceptability of a wearable technology physical activity intervention with telephone counseling for mid-aged and older adults: a randomized controlled Pilot trial. JMIR Mhealth Uhealth.

[140] Bailey DP, Harper JH, Kilbride C, McGowan LJ, Victor C, Brierley ML, Chater AM (2024). The frail-LESS (LEss sitting and sarcopenia in frail older adults) remote intervention to improve sarcopenia and maintain independent living via reductions in sedentary behaviour: findings from a randomised controlled feasibility trial. BMC Geriatr.

[141] Tunur T, DeBlois A, Yates-Horton E, Rickford K, Columna LA (2020). Augmented reality-based dance intervention for individuals with parkinson's disease: a pilot study. Disabil Health J.

[142] Widyastuti K, Makhabah DN, Setijadi AR, Sutanto YS, Ambrosino N, Suradi (2018). Benefits and costs of home pedometer assisted physical activity in patients with COPD. a preliminary randomized controlled trial. Pulmonology.

[143] Kidholm K, Ekeland AG, Jensen LK, Rasmussen J, Pedersen CD, Bowes A, Flottorp SA, Bech M (2012). A model for assessment of telemedicine applications: mast. Int J Technol Assess Health Care.

[144] VanSwearingen JM, Studenski SA (2014). Aging, motor skill, and the energy cost of walking: implications for the prevention and treatment of mobility decline in older persons. J Gerontol A Biol Sci Med Sci.

[145] Frechette ML, Meyer BM, Tulipani LJ, Gurchiek RD, McGinnis RS, Sosnoff JJ (2019). Next steps in wearable technology and community ambulation in multiple sclerosis. Curr Neurol Neurosci Rep.

[146] Woelfle T, Pless S, Wiencierz A, Kappos L, Naegelin Y, Lorscheider J (2021). Practice effects of mobile tests of cognition, dexterity, and mobility on patients with multiple sclerosis: data analysis of a smartphone-based observational study. J Med Internet Res.

[147] Salis F, Bertuletti S, Bonci T, Caruso M, Scott K, Alcock L, Buckley E, Gazit E, Hansen C, Schwickert L, Aminian K, Becker C, Brown P, Carsin A, Caulfield B, et al (2023). A multi-sensor wearable system for the assessment of diseased gait in real-world conditions. Front Bioeng Biotechnol.

[148] Low CA, Bartel C, Fedor J, Durica KC, Marchetti G, Rosso AL, Campbell G (2024). Associations between performance-based and patient-reported physical functioning and real-world mobile sensor metrics in older cancer survivors: A pilot study. J Geriatr Oncol.

[149] Mercer K, Giangregorio L, Schneider E, Chilana P, Li M, Grindrod K (2016). Acceptance of commercially available wearable activity trackers among adults aged over 50 and with chronic illness: a mixed-methods evaluation. JMIR Mhealth Uhealth.

[150] Loncar-Turukalo T, Zdravevski E, Machado da Silva J, Chouvarda I, Trajkovik V (2019). Literature on wearable technology for connected health: scoping review of research trends, advances, and barriers. J Med Internet Res.

[151] (2023). Digital Health Technologies for Remote Data Acquisition in Clinical Investigations - Draft Guidance for Industry, Investigators, and Other Stakeholders. United States Food and Drug Administration.

[152] (2020). Questions and answers: qualification of digital technology-based methodologies to support approval of medicinal products. European Medicines Agency.

[153] Walton MK, Cappelleri JC, Byrom B, Goldsack JC, Eremenco S, Harris D, Potero E, Patel N, Flood E, Daumer M (2020). Considerations for development of an evidence dossier to support the use of mobile sensor technology for clinical outcome assessments in clinical trials. Contemp Clin Trials.

[154] Goldsack JC, Coravos A, Bakker JP, Bent B, Dowling AV, Fitzer-Attas C, Godfrey A, Godino JG, Gujar N, Izmailova E, Manta C, Peterson B, Vandendriessche B, Wood WA, Wang KW, Dunn J (2020). Verification, analytical validation, and clinical validation (V3): the foundation of determining fit-for-purpose for biometric monitoring technologies (BioMeTs). NPJ Digit Med.

[155] Iaboni A, Spasojevic S, Newman K, Schindel Martin L, Wang A, Ye B, Mihailidis A, Khan SS (2022). Wearable multimodal sensors for the detection of behavioral and psychological symptoms of dementia using personalized machine learning models. Alzheimers Dement (Amst).

[156] Woelfle T, Bourguignon L, Lorscheider J, Kappos L, Naegelin Y, Jutzeler CR (2023). Wearable sensor technologies to assess motor functions in people with multiple sclerosis: systematic scoping review and perspective. J Med Internet Res.

[157] Zapletal A, Wells T, Russell E, Skinner MW (2023). On the triple exclusion of older adults during COVID-19: Technology, digital literacy and social isolation. Soc Sci Humanit Open.

[158] Sakal C, Li T, Li J, Yang C, Li X (2024). Association between sleep efficiency variability and cognition among older adults: cross-sectional accelerometer study. JMIR Aging.

