# Supplementary material for: Wearable Technologies for Health Promotion and Disease Prevention in Older Adults: Systematic Scoping Review and Evidence Map
Source: J Med Internet Res. 2025 Jun 24;27:e69077. doi: 10.2196/69077 (PMC12238792; doi:10.2196/69077)
Supplement: Multimedia Appendix 2 [file jmir_v27i1e69077_app2.docx]

**Appendix 2. PubMed search strategy**

#1 "wearable*"[Title/Abstract] OR "sensors"[Title/Abstract] OR "activity tracker*"[Title/Abstract] OR "garmin*"[Title/Abstract] OR "oura" OR [Title/Abstract] "acceleromet*"[Title/Abstract] "gyroscope"[Title/Abstract] OR “pedomet*" OR "smart watch*"[Title/Abstract] OR "smartwatch*"[Title/Abstract] OR "smart glass*"[Title/Abstract] OR "smartglass*"[Title/Abstract] OR "google glass*"[Title/Abstract] OR "fitness tracker*"[Title/Abstract]

#2 “Aged”[Mesh] OR "older adult"[Title/Abstract] OR "elder"[Title/Abstract] OR "ageing"[Title/Abstract] OR "aging"[Title/Abstract] OR "geriatric"[Title/Abstract]

#3 #1 AND #2
