# Supplementary material for: Wearable Technologies for Health Promotion and Disease Prevention in Older Adults: Systematic Scoping Review and Evidence Map
Source: J Med Internet Res. 2025 Jun 24;27:e69077. doi: 10.2196/69077 (PMC12238792; doi:10.2196/69077)
Supplement: Multimedia Appendix 3 [file jmir_v27i1e69077_app3.docx]

**Appendix 3. Extracted data form**

| **Extracted data** | **Definition** |
| --- | --- |
| **Study Characteristics** |  |
| Author | The first author of the study. |
| Year of publication | The year in which the study was published. |
| Country of publication | The country where the study was published. |
| Participants of study | The individuals who are included in the research. Their health status, whether they are healthy or have specific diseases. |
| Type of study design | The type of study design refers to the specific methodological approach used in a research study to collect and analyze data. |
| Number of participants | The number of participants from which the data was collected. |
| Mean age (range) | The mean/range age of the participants |
| Health condition | The health condition of the participants |
| **Wearable devices characteristics** |  |
| Measured biosignals | What are the biosignals measured by the wearable device (heart rate, EEG, ECG, step counts, body temperature, blood pressure, etc..)? |
| Sensors | What are the sensors embedded in the wearable device (Photoplethysmogram, accelerator, gyroscope)? |
| Sensing approach | Does the wearable device collect the data with the user’s input (i.e., manually adding data; participatory approach) or without the user’s input (i.e., automatic mechanisms for collecting data; opportunistic approach)? |
| Challenges and opportunities with wearable devices | What are the challenges and opportunities associated with wearable devices? |
| **AI Characteristics** |  |
| Use of algorithms | What are the main AI algorithms/models (e.g., RF, SVM, ANN, CNN, RNN, DNN, k-NN,) used in the paper? |
| Perfomance analysis of ML | What are the measures used to assess the performance of the algorithm (accuracy, sensitivity (recall), specificity, precision, AUC, etc...)? |
| Evaluation results | How are evaluations be conducted to involve end users in real-world settings(e.g., effectiveness, safety, cost, etc... )? |
| **Other** |  |
| Health target parameter | What specific health parameter or topic in the research？ |
